# Malignant transformation of oral potentially malignant disorders in males: a retrospective cohort study

**DOI:** 10.1186/1471-2407-9-260

**Published:** 2009-07-30

**Authors:** Pei-Shan Ho, Pai-Li Chen, Saman Warnakulasuriya, Tien-Yu Shieh, Yun-Kwan Chen, I-Yueh Huang

**Affiliations:** 1 Department of Oral Hygiene, College of Dental Medicine, Kaoshiung Medical University, Kaohsiung, Taiwan, Republic of China; 2 Department of Oral and Maxillofacial Surgery, Chiayi Christian Hospital, Chiayi, Taiwan, Republic of China; 3 Department of Oral Medicine & Pathology, King's College Dental Institute, WHO Collaborating Centre for Oral Cancer and Precancer, London, UK; 4 Division of Oral and Maxillofacial Surgery, Department of Dentistry, Kaoshiung Medical University Hospital, Kaohsiung, Taiwan, Republic of China; 5 Division of Oral Pathology, Department of Dentistry, Kaoshiung Medical University Hospital, Kaohsiung, Taiwan, Republic of China; 6 School of Dentistry, College of Dental Medicine, Kaoshiung Medical University, Kaohsiung, Taiwan, No.100 Shih-Chuan 1st Rd, Kaohsiung 807, Taiwan, Republic of China

## Abstract

**Background:**

Oral squamous cell carcinoma could be preceded by clinically evident oral potentially malignant disorders (OPMDs). Transformation of OPMDs to cancer has been studied in several population groups. It is difficult to undertake comparisons across populations due to variations in the methods of computation of malignancy rates among different studies. The aim of our study was to estimate the rate of malignant transformation of OPMDs taking into account the duration of follow-up and to identify the significant factors indicative of malignant potential.

**Methods:**

A total of 148 male patients with OPMDs were included. They were selected among all consecutive subjects registered at the maxillofacial clinic at a medical hospital in Kaohsiung, Taiwan. The mean follow up period was 37.8 months.

**Results:**

The malignant transformation rate was highest in subjects diagnosed with oral epithelial dysplasia. In this group the transformation rate was 7.62 per 100 persons-year. The rate in the group with verrucous hyperplasia (VH) was 5.21 per 100 persons-year, and in those with hyperkeratosis or epithelial hyperplasia was 3.26 per 100 persons-year. The anatomical site of OPMDs was the only statistically significant variable associated with malignancy. The hazard rate ratio (HRR) was 2.41 times for tongue lesions when compared with buccal lesions.

**Conclusion:**

The reported discrepancies of malignant transformation of OPMDs involve the follow-up time to cancer development and hence it is preferable to use a time-to-event estimation for comparisons. We found that malignant transformation of OPMDs involving the tongue was significantly higher than in other anatomical subsites after adjusting for the clinicopathological type or lifestyle factors at diagnosis.

## Background

Incidence of oral cancer has been rising in many countries in the world [1]. The 5-year survival rate for oral cancer has not significantly improved in the past 30 years and remains at approximately 50% [2]. Many oral squamous cell carcinomas are preceded by clinically evident oral potentially malignant disorders (OPMDs) [3]. It is very important to prevent malignant change in people diagnosed with OPMDs, but the hazard ratios of various OPMDs are not well known. The OPMDs include hyperkeratosis or epithelial hyperplasia, epithelial dysplasia [4-6], erythroplakia [7] and oral submucous fibrosis (OSF) [8,9] and their clinical phenotypes are well documented. hyperkeratosis or epithelial hyperplasia, epithelial dysplasia and OSF are the most common oral mucosal disorders in the regions where areca quid chewing is prevalent, such as India, Taiwan, and other Southeast Asian countries [9-15]. The malignant potential of oral lichen planus (OLP) remains controversial, because of the absence of universally accepted diagnosis criteria[16] therefore those diagnosed with OLP were not included in this study. The malignant transformation rates of OPMDs show a great variation; for example, 10–20% of hyperkeratosis or epithelial hyperplasia, epithelial dysplasia may transform to cancer and the estimated annual rate is 1.4%–7% [5,6,12,17]. In 1995, Lumerman reported that 6.6%–36% of epithelial dysplasias may transform to invasive SCC[18]. The studies concerning malignant transformation in OSF have been reported from India [8,9], with a reported malignant transformation rate of 2.3%–7.6% during 10–17 years of follow-up [8,9]. In a recent study in Taiwan [19], the malignant transformation rate of OPMDs was estimated at 3.02% in an average follow-up period of 42.6 months, and the transformation rate ranged from 1.9 to 5.4% for various types of OPMDs.

The malignant transformation of OPMDs to cancer has been studied in many different populations [5,6,8,9,12,17-19] and the evidence was reviewed by Napier & Speight [20]. It is difficult to undertake comparisons across populations due to variations in the methods of computation of malignancy rates among different studies. The malignant potential of a OPMDs should be a time-to-event function, not just calculated based on the number of malignant cases divided by total number of patients. Generally, a crude calculation by the event itself may contribute to bias [8,9,18]. This bias is due to the OPMDs patients with different follow-up durations, and clearly longer follow-up periods contribute to higher transformation percentages. Hence, a person-time malignant transformation rate is more accurate in terms of cross population comparisons.

The risk factors associated with these OPMDs and oral cancer have been established. Tobacco use, alcohol abuse and areca quid chewing habits are important risk factors. The associated factors in the progression of the disease and malignant transformation of OPMDs have not been well defined in previous studies. Lesion type [5,6,8,9,12,17-19], age [6,21], lifestyle habits [5,6,22] and lesion subsites [23,24] were significant factors related to malignancy, but the results from different studies vary and firm conclusions cannot be drawn.

The aim of our study was to estimate the malignant transformation rate by taking into account the varying individual lengths of follow-up of a cohort of consecutive subjects diagnosed with OPMDs in Taiwan and to identify the significant factors contributing to malignant potential of OPMDs using a time-to-event analysis method.

## Methods

In this hospital-based follow-up study a total of 148 male subjects with OPMDs were included. We searched the files of all patients admitted at the maxillofacial clinic at a medical hospital in Kaohsiung, Taiwan from 1986 to 2004. The consecutive subjects with clinicopathological diagnosis of OPMDs, which including hyperkeratosis or epithelial hyperplasia, epithelial dysplasia, verrucous hyperplasia and oral submucous fibrosis (OSF). The exclusion criteria were:

1. Any patient with OPMD who had not been followed-up for more than 6 months

2. Those patients who had oral cancer before the diagnosis of OPMDs.

3. The patients who were ever diagnosed as OPMDs and received prior medical therapy.

4. The duration from OPMDs to oral cancer was less than 6 months.

No medical or surgical treatments were offered to this group except habit interventions and repeat biopsy when indicated clinically during follow up.

The diagnosis of OPMDs and development of SCC during follow-up were confirmed by histopathology. These histological diagnoses used here were described in more detail in a previous report [19]. The diagnostic criteria for the detection of OPMDs were based on the recommendations of the WHO [25,26] and the First International Seminar on 'Oral Leukoplakia'[27]. Verrucous hyperplasia(VH) is rarely seen in western countries, but is a common type of OPMD in Taiwan. Yu's [28], described the histological criteria for a diagnosis of verrucous hyperplasia: (1) epithelial hyperplasia with parakeratosis or hyperkeratosis and a verrucous surface, and (2) no invasion of the hyperplastic epithelium into the lamina propria as compared to adjacent normal mucosal epithelium.

During analysis of data on subjects with multiple lesions, we considered the first diagnosed lesion as the index lesion. The primary end point of the study was development of oral cancer in a existing OPMDs. Each subject had regular follow-up at approximately three-monthly intervals during the study period.

The information on lifestyle habits, including smoking, use of alcohol, areca quid and site by lesions were collated from the clinic records. These data were recorded prospectively during the patients' first visit to our clinic.

Chi-square test was used to compare the distribution of related factors in OPMDs and in the group with malignancies. Time-to-event analysis involved estimating the probability that an event will occur at different points in time. The end point of follow-up in those developing cancer was the date of detection of oral malignancy, and in those lost to follow up were coded by the date of last visit, to arrive at "censored" data. The most common time-to-event statistical method is the Kaplan-Meier method and the Cox proportional hazard model. The Kaplan-Meier estimate was computed to estimate the probability of cancer-free survival. The Cox proportional hazards model was applied to analyze the effect of single and multiple covariates in predicting cancer development.

This study complied with the Helsinki Declaration. The data in this study were collected after the approval of the Institutional Review Board of Kaoshiung Medical University Hospital (KMUH-IRB-970157) after obtaining informed consent.

## Results

The mean follow-up period of the cohort of 148 male patients was 37.8 months. Among these subjects. Buccal, vestibule and retromolar areas were the most common locations of these OPMDs, and there were 100 patients (65.8%) with lesions located in these subsites. There were 21 subjects (13.8%) with lesions on the tongue and 18 (11.8%) on the lip. Histological examination of the initial biopsy revealed 67 subjects (42.3%) with hyperkeratosis or epithelial hyperplasia, this being the most common pathology diagnosis among the study group. Verrucous hyperplasia (VH) was the second most common lesion, with 44 subjects (29.7%) in this study. Thirty three subjects had epithelial dysplasia (22.3%) and 4 had OSF (2.7%).

The profile of the 4 groups showing characteristics of malignant transformation is shown in Table 1. The malignant transformation rate was highest in the group with epithelial dysplasia (24.2%), followed by verrucous hyperplasia (20.0%), and hyperkeratosis or epithelial hyperplasia (8.6%). In Table 1, the person-time malignant rate was also estimated with consideration given to the duration of follow-up. The rate of malignant transformation for those with epithelial dysplasia was 7.6 per 100 persons-year, 5.2 per 100 persons-year in VH, and 3.3 per 100 persons-year in those with hyperkeratosis or epithelial hyperplasia. In our study, none of the subjects with OSF had malignant change during the follow-up period.

**Table 1:** Malignant Transformation in OPMDs

	Age(years)^1^	Mean Duration(months)^2^	Duration Range^3^	Malignancy(%)^4^	Transformation rate(CI)^5^
Epithelial dysplasia	46.70(11.51)	38.18(27.90)	6.53–101.2	8 (24.24)	7.62 (2.34–12.90)
Verrucous hyperplasia	46.66(11.11)	41.87(42.47)	6.07–185.5	9 (20.00)	5.21 (1.60–8.82)
Hyperkeratosis or epithelial hyperplasia	42.51(12.98)	32.94(32.81)	6.13–135.23	6 (8.57)	3.26 (0.65–5.87)
OSF	39.50(21.05)	71.13(50.45)	27.27–140.97	0 (0.00)	-

^1 ^The average age with standard deviation at first diagnosis

^2 ^The average follow-up duration with standard deviation before malignant event

^3 ^The range of follow-up duration before malignant event

^4 ^The number and proportion of malignant transformation

^5 ^The malignant transformation rate with 95% Confidence Interval (100 persons-year)

Table 2 shows subject and lifestyle factors related to malignant transformation in OPMDs. Those older than 45 years of age at their first diagnosis showed a slightly higher malignant potential than the younger group at first diagnosis (20.0% vs 11.4%), but age at diagnosis was not statistically significant. The absence of smoking or chewing habit seemed to have a higher risk for developing malignancy. The development of malignancy in non-smokers or non-chewers with OPMDs was about 40%, which was approximately 3-fold higher than for smokers or chewers. About 50% of lesions located on the tongue (11/22) were found to transform, and the rate was significantly higher than lesions located on other subsites (P < 0.0001).

**Table 2:** The Factors Related to Development of Malignancy of OPMDs(n = 148)^1^

	Malignant Transformation				
		
	Yes		No		
		
	N	%	N	%	P value
Age					
<=45 years	13	20.00	52	80.00	
> 45 years	9	11.39	70	88.61	0.1531
Smoking					
No	18	13.64	114	86.36	0.0447
Yes	4	36.36	7	63.64	
Alcohol use					
No	13	14.13	79	85.87	0.7273
Yes	8	16.33	41	83.67	
Areca quid use					
No	20	14.49	118	85.51	0.1204
Yes	2	40.00	3	60.00	
Location					
Buccal^2^	8	8.25	89	91.75	< 0.0001
Tongue	11	52.38	10	47.62	
Other^3^	3	11.54	23	88.46	

^1^: OSF cases are excluded in this table

^2^: Including buccal, vestibule and retromolar

^3^: Including lip, gingiva, palate

**Figure 1:**
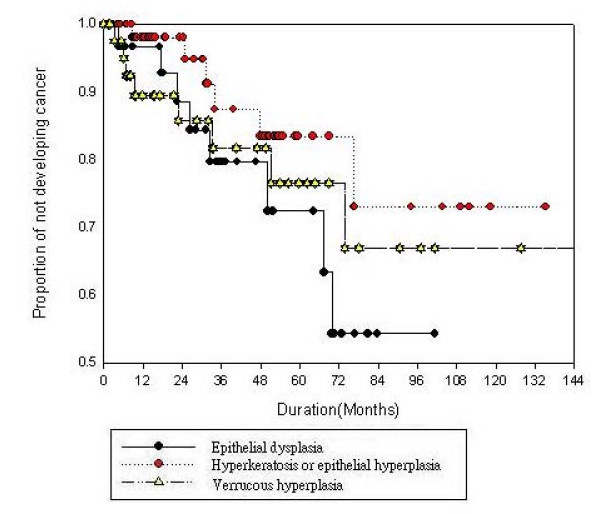
**The Kaplan-Meir curve for cancer free survival by OPMDs**.

Figure 1 shows the Kaplan-Meier analysis for cancer-free survival by different OPMDs. From this analysis, we found that epithelial dysplasia and VH showed similar patterns of cancer-free survival. A rapid descending curve was discovered in the first 2–3 years in all OPMDs. The Kaplan-Meier estimates of the 3-year cancer-free rate of epithelial dysplasia and VH were less than 80%, which meant that the malignant rate of these two OPMDs were higher than 20%. The 3-year malignant transformation rate of hyperkeratosis or epithelial hyperplasia was approximately 10%. Epithelial dysplasia and VH seemed to show higher malignant potential than hyperkeratosis or epithelial hyperplasia. Further investigation with Cox proportional hazard regression analysis was performed (Table 3). In the multiple regression models, we considered the effect of age of diagnosis, gender, subsite location, lesion type and lifestyle habits on malignant transformation. We found that after adjusting for other factors, lesions located on the tongue were found to have a higher malignant risk, with a hazard ratio (HRR) of 2.41 (1.43, 4.16) when compared to lesions located on the buccal mucosa. In addition to subsite location, age at diagnosis and lesion type were also important factors related to malignancy. Presence of epithelial dysplasia and VH showed higher malignant risk than hyperkeratosis or epithelial hyperplasia. HRRs were 1.51 times in epithelial dysplasia and 1.23 times in VH. The malignant risk for the group diagnosed before 45 years of age was 1.42 times more than those below 45 years. But the age at diagnosis and type of OPMD were not with statistically significant largely due to the small sample size.

**Table 3:** Proportional Hazards Model of Malignant Transformation for Related Factors(n = 148)^1^

Variable		RR	95%CI	P value	ARR	95%CI	P value
Age	<=45 years	1.00			1.00		
	> 45 years	1.42	(0.93,2.22)	0.1007	1.42	(0.90,2.31)	0.1184
							
OPMDs	Hyperkeratosis or epithelial hyperplasia	1.00			1.00		
	Verrucous hyperplasia	1.38	(0.81,2.44)	0.2304	1.23	(0.66,2.31)	0.5077
	Epithelial dysplasia	1.56	(0.92,2.82)	0.0991	1.51	(0.86,2.73)	0.1493
							
Location	Buccal^2^	1.00			1.00		
	Tongue	2.31	(1.46,3.71)	< 0.001	2.41	(1.43,4.16)	0.0012
	Other^3^	1.34	(0.62,2.50)	0.4103	1.37	(0.62,2.68)	0.4062
							
Smoking	No	1.00			1.00		
	Yes	0.77	(0.47,1.44)	0.3958	0.98	(0.48,2.31)	0.9556
							
Alcohol use	No	1.00			1.00		
	Yes	0.99	(0.65,1.58)	0.9809	1.10	(0.68,1.87)	0.7134
							
Areca quid use	No	1.00			1.00		
	Yes	0.53	(0.28,1.34)	0.1505	0.98	(0.36,2.97)	0.9682

^1^: OSF cases are excluded in this table

^2^: Including buccal, vestibule and retromolar

^3^: Including lip, gingival, palate

## Discussion

The present study describes some general features of OPMDs in a hospital outpatient population in Taiwan. The buccal, vestibule and retromolar areas were the most common sites affected. These together accounted for more than 60% of affected sites. Hyperkeratosis or epithelial hyperplasia, epithelial dysplasia was the most common OPMD. About 45% of OPMDs were epithelial dysplasia and 30% had epithelial dysplasia. In the US, 85% of OPMDs are hyperkeratosis or epithelial hyperplasia, epithelial dysplasia, and more than two thirds of all oral hyperkeratosis or epithelial hyperplasia, epithelial dysplasia are found on the lip, buccal mucosa and gingiva. In most studies from western countries, the predominant OPMDs sites are the tongue and floor of the mouth[5,6,29,30]. But studies from India, Taiwan and other Southeast Asian countries, indicated that the buccal mucosa is the most common site of premalignancy [9-11,13,14]. It is suggested that the differences of site distribution and lesion type may be related to different risk factors in different populations [10,11]. In this study, we found the mean age at first dignosis of OPMDs was about 45 years. A young age at first diagnosis of OPMDs has also been reported in previous studies of Taiwan [11,31]. In comparison the mean age of diagnosis of hyperkeratosis or epithelial hyperplasia, epithelial dysplasia in reports from other countries is about 55–60 years [6,18,32]. It is suggested that OPMDs occurring at a younger age may be related to subsequent development of oral cancer while they are still young. This is consistent with the mean age at diagnosis of oral cancer in Taiwan is about 10 years younger than in other countries[33].

In the present study, we compared malignant potential of several common OPMDs. We also took follow-up duration into account to estimate the malignant transformation rate and to evaluate related factors of malignancy. In previous studies, the malignant transformation rate of hyperkeratosis or epithelial hyperplasia, epithelial dysplasia was reported to be 10–20% and the annual rate was 1.4%–7% [5,6,12,17]. In our study, hyperkeratosis or epithelial hyperplasia, epithelial dysplasia had a malignant transformation rate from 8% to 24%, and the annual rate is 3–8 per 100 persons-year. Epithelial dysplasia have the highest transformation rate, and this is consistent with previous reports [18,24].

VH has rarely been reported in scientific literature and the malignant potential of VH has not been examined in detail. In the hospital clinics in Taiwan, VH has been noted in areca quid chewers [15]. The annual malignant transformation rate of VH is about 5.2 per100 persons-year, and almost 1.5-fold higher than hyperkeratosis or epithelial hyperplasia. In the context of Taiwan VH appears an important entitity among OPMDs [19]. The transformation rate of VH in our study is higher than in Hsue's study [19]. In this study, none of the OSF patients showed evidence of malignant transformation. This is probably because of the small number of patients with OSF as the first OPMD diagnosis. OSF is a common comobidity lesion, which is often found coexisting with other OPMDs [9]. In the present study, few subjects were first diagnosed as OSF. Due to low prevalence a larger cohort is needed to examine the malignant profile of OSF.

To compare different malignant transformation patterns in OPMDs, a follow-up curve was plotted according to the Kaplan-Meier method. Differences were noted in the three types of OPMDs entered in the study. During the first 2–3 years of follow-up a higher risk of malignant transformation was noted, an observation that was also found in Silverman's study [6]. It is suggested that rigorous follow-up in the first two years after diagnosing OPMDs may be important in detecting early occurrence of malignancy [6]. The higher risk observed during the earlier phases of follow-up could be due to sampling errors of biopsy diagnosis of OPMDs. This needs to be verified in further studies. It is proposed that studies on the natural history should exclude any cases transforming within 6 months of biopsy to allow for sampling errors.

Lesions located on the tongue may have a higher malignant risk, which has also been reported in other studies[23,24]. Schepman's study, however, showed that there were no particular oral subsites associated with an increased risk of oral cancer [5]. The subsite variation in malignant transformation may need further study. Age is also an important factor affecting malignant transformation, which may be related to genetic susceptibility contributing to the phenotype. In studies by Bouquot [21] and Silverman et al[6], it was reported that the longer the existence of hyperkeratosis or epithelial hyperplasia, epithelial dysplasia the worse its prognosis.

Shiu et al.'s study in 2000 indicated that betel quid chewing is a significant factor influencing malignant transformation in hyperkeratosis or epithelial hyperplasia, epithelial dysplasia[22]. In Schepman's study in 1998 and Silverman's in 1984 [5,6], the absence of smoking was associated with an increased risk of malignancy in hyperkeratosis or epithelial hyperplasia, epithelial dysplasia. Our findings also confirmed a lack of effect of smoking, drinking and chewing on OPMDs malignant change, as these factors were not significant in our study. It may be said that lifestyle habits play significant roles in the development of OPMDs, but one can not state with certainty that these habits promote malignant transformation of OPMDs[23]. The lifestyle habits collected in our study were from the case records of the patients' first visit, and the prevalence of these habits may be under reported. The risk factors relating to malignant formation of OPMDs were not well defined in previous studies. In the present data, we found that an age older than 45 years, lesions on the tongue, epithelial dysplasia and VH might be important risk factors related to malignancy. The lifestyle habits may be associated with the development of OPMDs, they do not appear to affect the prognosis. This may be because some subjects quit their risk habits following the detection of an OPMD. Further study is needed to clarify the trend of lifestyle habits following diagnosis of OPMDs, and the roles of intervention in the malignant process of OPMDs.

Significant gender differences were found in oral cancer cases and OPMDs of Taiwan[10,19,33]. To avoid the confusing etiological effect between genders, only males were included in the present study.

## Conclusion

In conclusion, the importance of certain risk factors, such as oral subsite, age at diagnosis and type of OPMD that are associated with an increased chance of malignant transformation of OPMDs in Taiwan are reported here. The malignant potential of epithelial dysplasia and VH is much higher than that of hyperkeratosis or epithelial hyperplasia. Rigorous follow-up is advised in the first 2–3 years after the detection of OPMDs. Finding a lesion located on the tongue appears to be the most important factor affecting malignant transformation of OPMDs.

## Competing interests

The authors declare that they have no competing interests.

## Authors' contributions

PSH and PLC conducted, designed, implement and drafted the manuscript. SW gave important comments to the original draft of manuscript. TYS helped to review the manuscript. YKC helped to confirm the histopathology diagnosis in these OPMDs and oral cancer lesions. IYH conceived the study, and participated in its design and coordination and helped to draft the manuscript. All authors read and approved the final manuscript.

## Pre-publication history

The pre-publication history for this paper can be accessed here:

http://www.biomedcentral.com/1471-2407/9/260/prepub
